# MiR-146a rs2910164 polymorphism and head and neck carcinoma risk: a meta-analysis based on 10 case-control studies

**DOI:** 10.18632/oncotarget.13599

**Published:** 2016-11-25

**Authors:** Zhaolan Xiang, Jue Song, Xianlu Zhuo, Qi Li, Xueyuan Zhang

**Affiliations:** ^1^ Department of Otolaryngology, Southwest Hospital, Third Military Medical University, Chongqing, China; ^2^ Affiliated Hospital of Guizhou Medical University, Guiyang, China

**Keywords:** miR-146a, head & neck cancer, susceptibility, meta-analysis, polymorphism

## Abstract

Two recent meta-analyses have been conducted on the relationship between miR-146a polymorphism (rs2910164) and head and neck cancer (HNC) risk. However, they have yielded conflicting results. Hence, the aim of the present study was to conduct a quantitative updated meta-analysis addressing this subject. Eligible studies up to Sep 2016 were retrieved and screened from the bio-databases and then essential data were extracted for data analysis. Next, subgroup analyses on ethnicity, source of controls, sample size, and genotyping method were also carried out. As a result, a total of 9 publications involving 10 independent case-control studies were included. The overall data indicated a significant association between miR-146a rs2910164 polymorphism and HNC risk [C vs. G: odds ratio (OR) = 1.14; 95% confidence interval (CI) = 1.00–1.31; CC+CG vs. GG: OR=1.21; 95%CI=1.02-1.43]. Variant alleles of miR-146a rs2910164 may have a correlation with increased HNC risk. Future well-designed studies containing large sample sizes are needed to verify this result.

## INTRODUCTION

Head and neck carcinoma (HNC) has ranked the sixth most frequent malignancy worldwide, which comprises a number of epithelial cancers originated from oral, nasal cavity, pharynx and larynx [1]. The occurrence of HNC shows a decreasing trend and its mobility is still high in spite of receiving comprehensive treatment involving radiation, chemotherapy, and surgical treatment modalities [2]. Life qualities of patients can be seriously affected by HNC due to its specific sites that have an association with speaking and breathing [3].

The etiology of HNC remains largely unclear. In recent years, microRNAs (miRNAs) have attracted much attention. MiRNAs are a series of single-stranded short non-coding RNAs that can inhibit gene expression by directly targeting specific mRNAs. They have been suggested to be involved in cellular processes such as cell proliferation, differentiation and apoptosis [4]. Thus, aberrant expressions of miRNAs have been indicated to have a correlation with etiology, diagnosis and development of many cancers. MiRNAs function as either tumor suppressors or oncogenes in HNC [5]. Single nucleotide polymorphism is a variation of DNA sequence, which occurs in a proportion of population. The variation of miRNAs might interfere with the translation of mRNA at the post-transcriptional level and suppress gene expression, thus leading to abnormal biological metabolism and cellular process [6].

A popular miRNA, miR-146a, has been suggested to have an association with the development of a variety of disorders [7]. It also functions as an oncogene or a tumor suppressor in various cancers [8]. A polymorphism of miR-146a that is located on chromosome 5q34 with a nucleotide mutation from G to C (rs2910164) has been reported to be related with a number of cancers [9]. Previously, a growing body of published literature has been devoted to the relationship between miR-146a rs2910164 polymorphism and HNC risk. Nevertheless, the results were conflicting. Hence, in 2015, two meta-analyses addressing this topic have been published [10, 11]. However, interestingly, the results of these two meta-analyses were conflicting. On the basis of this situation, we aimed to conduct an updated meta-analysis including recently published data up to Sep 2016 to derive a more precise estimation of the relationship.

## RESULTS

### Study characteristics

**Figure 1:**
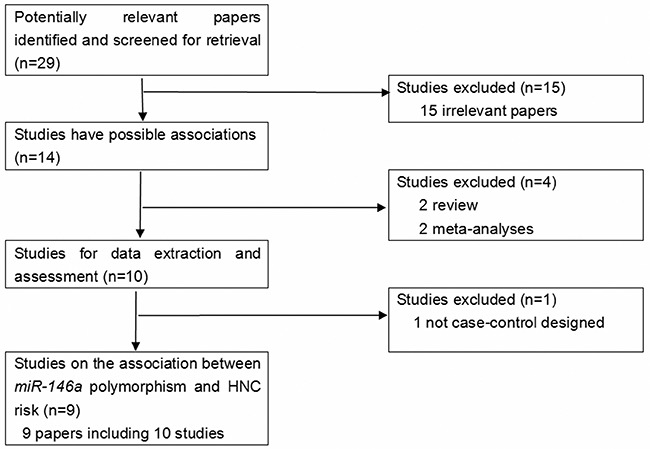
The flow diagram of included/excluded studies

Potential publications were retrieved from the databases. A total of 29 publications were originally obtained, among which 15 irrelevant papers were firstly excluded. Thus, 14 publications were eligible. Then, 2 review articles [12, 13] and 2 meta-analyses on the same topic [10, 11] were discarded. Next, 1 study that was not case-control designed [14] was also eliminated. As a result, 9 publications were selected for data extraction and assessment. Notably, one paper by Chen et al. [15] contained two independent studies. Therefore, there were 9 papers [15–23] that contained 10 case-control studies in the present meta-analysis (Figure 1).

All publications were written in English, except for one in Chinese [23]. The relevant information of the selected papers such as the first author, the number and characteristics of cases and controls for each study was listed in Table 1. There were 3 groups of Caucasians [16, 19, 21] and 7 groups of Asians [15, 17, 18, 20, 22, 23] in the present analysis. The distribution of the miR-146a rs2910164 genotypes and the genotyping methods of the included studies were presented in Table 2. The genetic distributions of the control groups in all studies were all consistent with the HWE.

**Table 1:** Characteristics of studies included in the meta-analysis

First Author	Publication Year	Number of Cases (male/female)	Number of Controls (male/female)	Type of controls	Matches of controls	Median (or mean) age, year (Cases/Controls)	Racial decent	Type	Country
Liu	2010	1109 (837/272)	1130 (860/270)	Healthy controls (PB)	Age, sex	57.2/56.8	Caucasian	Combined	USA
Chu	2012	470 (470/0)	425 (425/0)	Healthy controls (PB)	NA	NA/NA	Asian	Mouth	China
Lung	2013	233 (172/61)	3613 (1769/1844)	Healthy controls (PB)	NA	51.3/70.3	Asian	Nasopharynx	China
Orsos	2013	468 (362/106)	468 (362/106)	Cancer-free patients (HB)	Age, sex, smoking	65.3/64.8	Caucasian	Combined	Hungary
Huang	2014	160 (117/43)	200 (132/68)	Cancer-free patients (HB)	Age, sex	46.2/44.7	Asian	Nasopharynx	China
Lin	2014	204 (199/5)	440 (434/6)	Healthy controls (PB)	NA	61.4/62.1	Asian	Larynx	China
Palmieri	2014	346 (252/94)	88 (NA/NA)	Healthy controls (PB)	NA	65.0/NA	Caucasian	Combined	Italy
Chen (1)	2016	188 (NA/NA)	197 (NA/NA)	Cancer-free patients (HB)	Age, betel chewing	47.7/46.9	Asian	Combined	China
Chen (2)	2016	658 (618/40)	668 (628/40)	Cancer-free patients (HB)	Age, sex	52.8/52.8	Asian	Combined	China
Miao	2016	576 (362/214)	1552 (987/565)	Cancer-free patients (HB)	Age, sex	NA/NA	Asian	Combined	China

NA: not available; PB: population-based; HB: hospital-based

**Table 2:** Distribution of miR-146a rs2910164 polymorphism genotypes among HNC cases and controls included in the meta-analysis

First Author	year	Genotyping method	Cases			Controls			HWE (control)	
			CC	CG	GG	CC	CG	GG	Chi-square	P
Liu	2010	PCR-RFLP	68	411	630	70	405	655	0.486	> 0.05
Chu	2012	PCR-RFLP	174	242	54	175	196	54	0.006	> 0.05
Lung	2013	PCR	117	88	24	1413	1721	479	1.577	> 0.05
Orsos	2013	PCR	16	168	284	9	136	323	1.522	> 0.05
Huang	2014	PCR-RFLP	64	73	23	54	110	36	2.375	> 0.05
Lin	2014	Taqman	63	110	31	81	220	139	0.138	> 0.05
Palmieri	2014	Taqman	19	121	197	7	31	50	0.488	> 0.05
Chen (1)	2016	Taqman	73	84	31	80	82	35	2.917	> 0.05
Chen (2)	2016	Taqman	253	318	87	272	293	103	2.632	> 0.05
Miao	2016	Illumina	101	281	194	278	773	497	0.565	> 0.05

### Meta-analysis results

The main results of the meta-analysis are listed in Table 3. The heterogeneity in the allelic contrast (P=0.000), dominant model (P=0.007) and recessive model (P=0.000) was significant, respectively. Therefore, the random-effect models were used for calculation in these models.

**Table 3:** Main results of the pooled data in the meta-analysis

	No. of studies	C vs G			(CC +CG) vs GG			CC vs (CG +GG)		
		OR (95%CI)	P	P (Q-test)	OR (95%CI)	P	P (Q-test)	OR (95%CI)	P	P (Q-test)
Total	10	1.14 (1.00-1.31)	0.047	0.000	1.21 (1.02-1.43)	0.025	0.007	1.16 (0.94-1.44)	0.168	0.000
Ethnicity										
Caucasian	3	1.11 (0.88-1.39)	0.383	0.062	1.15 (0.90-1.46)	0.267	0.103	1.05 (0.70-1.56)	0.821	0.276
Asian	7	1.16 (0.97-1.39)	0.094	0.000	1.27 (0.99-1.63)	0.065	0.006	1.19 (0.92-1.54)	0.174	0.000
Source of controls										
PB	5	1.18 (0.93-1.49)	0.178	0.000	1.28 (0.93-1.76)	0.126	0.004	1.19 (0.82-1.73)	0.356	0.000
HB	5	1.10 (0.95-1.29)	0.212	0.025	1.16 (0.95-1.41)	0.146	0.134	1.10 (0.86-1.41)	0.456	0.049
Sample size										
≤1000	4	1.07 (0.93-1.22)	0.361	0.025	1.04 (0.93-1.17)	0.463	0.393	1.09 (0.83-1.43)	0.529	0.007
<1000	6	1.20 (0.96-1.52)	0.115	0.000	1.35 (1.02-1.78)	0.034	0.028	1.24 (0.85-1.82)	0.263	0.001
Genotyping method										
PCR-RFLP	3	1.07 (0.89-1.29)	0.469	0.080	1.07 (0.93-1.25)	0.347	0.748	1.11 (0.74-1.67)	0.622	0.015
PCR	2	1.39 (1.19-1.62)	0.000	0.945	1.40 (1.12-1.76)	0.004	0.700	1.64 (1.27-2.12)	0.000	0.814
Taqman	4	1.13 (0.83-1.56)	0.433	0.000	1.34 (0.87-2.06)	0.180	0.007	1.09 (0.71-1.67)	0.692	0.004
Illunima	1	0.96 (0.84-1.10)	0.558	-	0.93 (0.76-1.14)	0.490	-	0.97 (0.76-1.25)	0.821	-

PB: population-based; HB: hospital-based

**Figure 2:**
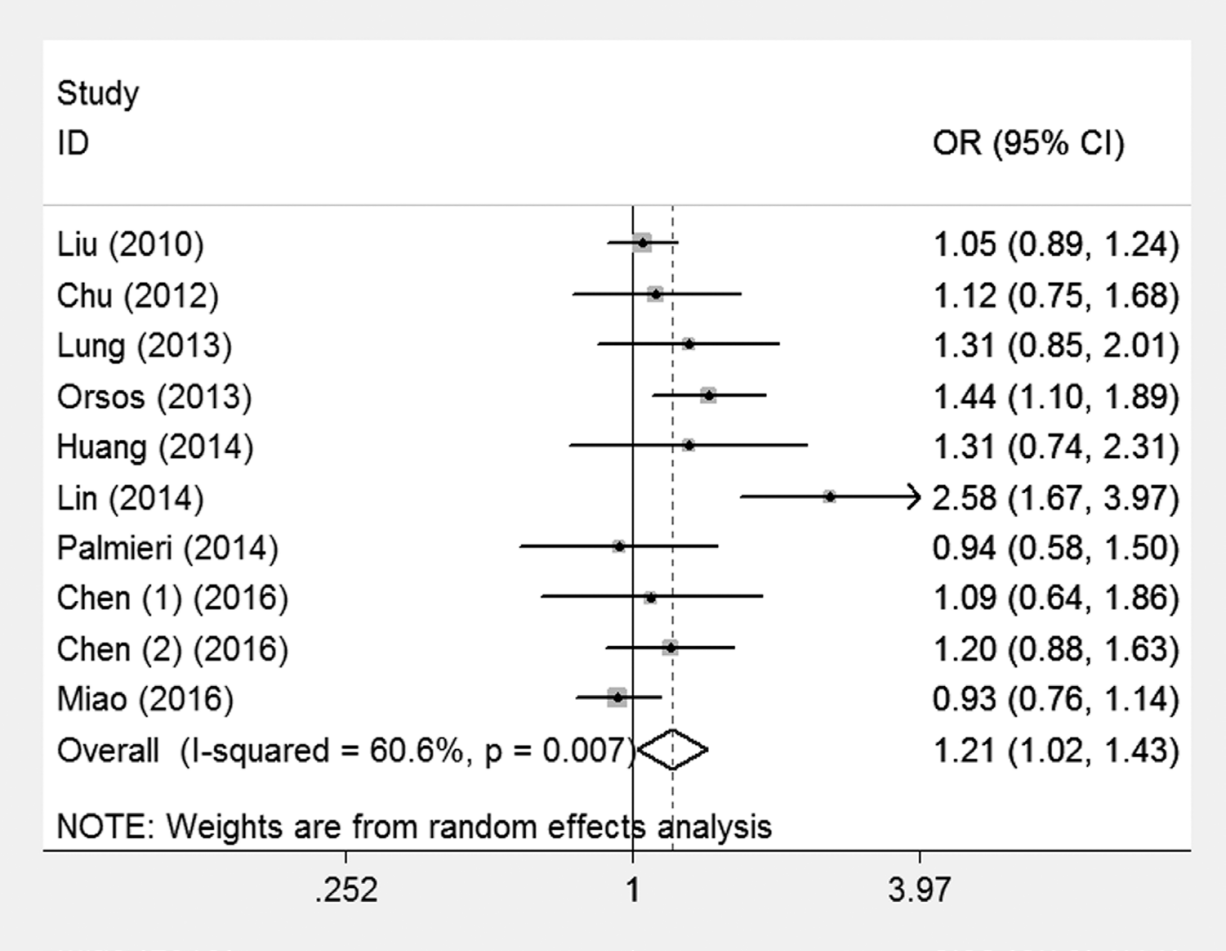
Meta-analysis for the association of HNC risk with miR-146a rs2910164 polymorphism for the overall data (CC+CG vs GG)

For the overall data, a total of ten case-control studies containing 4399 cases and 8777 controls were involved. The pooled ORs for the recessive model (OR=1.16; 95% CI = 0.94-1.44) failed to show an association. Nevertheless, borderline increased cancer risks could be shown in both the allelic contrast (OR=1.14; 95% CI = 1.00-1.31) and dominant model (OR=1.21; 95% CI =1.02-1.43), implying that variant C allele of miR-146a rs2910164 may have a correlation with increased risk of HNC (Figure 2).

To evaluate the effect of confounding factors on the results, we conducted subgroup analyses according to ethnicity, source of controls, sample size, and genotyping method, respectively. However, no association could be observed in most of the subgroups, except for the subgroup analysis on ‘PCR’, and ‘<1000’ under the dominant model.

### Sensitivity analysis and bias diagnostics

To test the stability of the overall results, we firstly changed the effect models and found that the results were not statistically altered. Then, we deleted one study from the database each time and repeated the analyses. The data showed that the overall results were not statistically changed in the above analysis, indicating that the overall results of the present study were stable.

**Figure 3:**
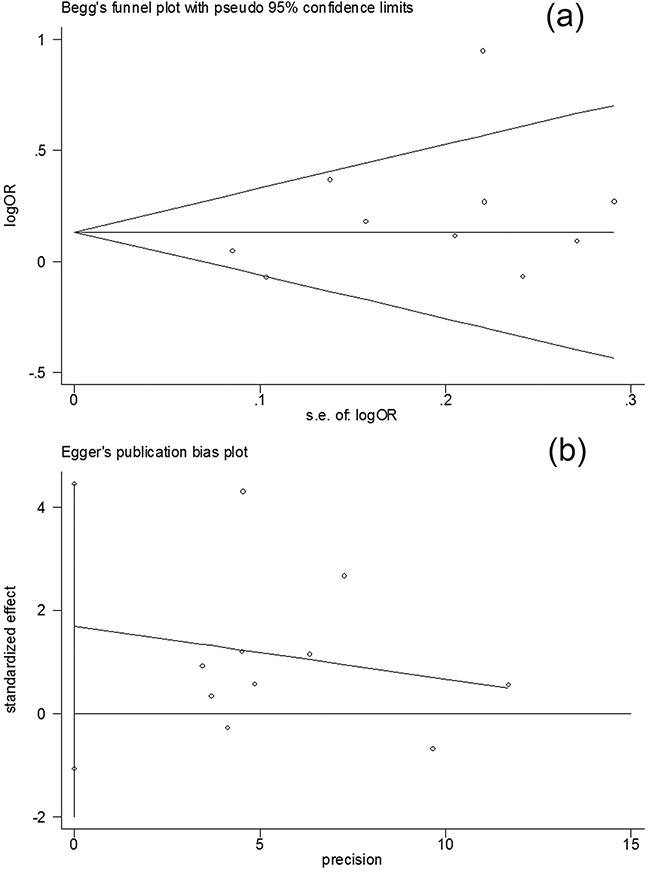
Publication bias test for the overall data (CC+CG vs GG) **a.** Funnel plot; **b.** Egger's linear regression test.

Publication bias was an unavoidable problem in the meta-analysis. Funnel plots were generated and their symmetries were further evaluated by Egger's linear regression tests. As expected, the data showed that the plots for the three genetic models were symmetrical (C vs. G: *t* = 0.82, *P* =0.437; dominant model: *t* = 1.41, *P* =0.196; recessive model: *t* = 0.82, *P* =0.437), suggesting that the publication bias was not evident (Figure 3).

## DISCUSSION

MiR-146a rs2910164 polymorphism may play different roles in the susceptibility to different cancers. For HNC, two meta-analyses published in 2015 had concerned the relationship between miR-146a rs2910164 polymorphism and HNC risk. However, the results were inconclusive. The study by Chen et al. [10] failed to show an association between miR-146a rs2910164 polymorphism and HNC risk, while conversely, the study by Niu et al. [11] showed that rs2910164 of miR-146a confers HNC risk among Caucasians. It is worthy of note that several problems might exist in the above two meta-analyses. In the paper by Chen et al. [10], a study [14] that was not case-control designed had been regarded as case-control study and thus were selected. This error processing might lower the credibility of the overall results. For the meta-analysis by Niu et al. [11], the data of thyroid cancer had been combined as HNC in the database. However, both the pathology and biological behavior of thyroid cancers are obviously different from other HNCs. Thus, inclusion of thyroid cancers in HNC series for this type of meta-analysis might reduce the power to evaluate the association.

In the present study, we found that miR-146a rs2910164 polymorphism might have a relationship with HNC risk. However, the association could not be observed when the data were stratified by ethnicity, inconsistent with the published two meta-analyses [10] [11]. This might be due to the ethnic variation or the limited number of the included studies.

The mechanisms by which miR-146a rs2910164 polymorphism increases HNC risk were not fully understood. Previous reports have shown that polymorphism might result in down-regulation of miR-146a expression [24], which may have a correlation with distant metastasis of HNC [25]. In some cancers, miR-146a acts as a tumor suppressor. For instance, in a study on breast cancer, activated miR-146a may attenuate epidermal growth factor receptor expression, thus influencing the disease progression, and clinical prognosis [26]. In addition, miR-146a can inhibit epithelial mesenchymal transition and thus suppress lung cancer progression [27]. Therefore, inhibition of miR-146a expression may have an association with cancer risk. This might help clarify the reason why miR-146a rs2910164 polymorphism has a relation to increased HNC risk.

Several limitations need to be addressed in this meta-analysis. First, only English and Chinese were used in the search strategy. Thus, articles written in other languages were missed in the searching process, leading to any selection bias. Second, only Asian and Caucasian populations were involved in the present study. Other ethnicities such as African were not included. Since gene variations might be different in different ethnicities, future studies on various ethnicities are needed.

In conclusion, the overall data reveal a significant association of miR-146a rs2910164 polymorphism with HNC risk, and nevertheless, future studies on different ethnicities with large sample sizes are needed to obtain a more convincing result.

## MATERIALS AND METHODS

### Ethics statement

Ethical approval is not necessary for the present meta-analysis.

### Literature search strategy

A systematic search was conducted in the databases such as Medline, EMBASE, Web of Science and Chinese National Knowledge Infrastructure (CNKI). Published papers up to Sep 2016 were covered. The following keywords were used: *miRNA*, *miR-146a*, *oral*, *mouth*, *laryngx, pharyngx, nasopharynx, head and neck, neoplasm*, *cancer*, *variation*, and *polymorphism*. All potential studies were retrieved and the bibliographies were further checked for possible publications whenever necessary.

### Inclusion criteria

For the literature inclusion, the following criteria were used: (1) papers should concern miR-146a rs2910164 polymorphism and HNC risk; (2) studies should be case-control designed; (3) papers should state adequate information for readers to calculate odds ratios (ORs) and their 95% confidence intervals (CIs). Accordingly, the following criteria were used for exclusion: (1) duplicate publication; (2) papers with insufficient information.

### Data extraction

The data were extracted by two of the authors independently. If the extracted information was conflicting, a discussion was conducted to reach an agreement. If the agreement could not be reached, another author was consulted and a final decision was made according to the majority of votes. When two or more studies shared the same group of population, only the study including the larger or the largest sample size was selected.

### Statistical analysis

The Hardy-Weinberg equilibrium (HWE) was assessed by Fisher's exact test for the controls in each study. The ORs and their 95%CIs were calculated to evaluate the strength of the association between miR-146a rs2910164 polymorphism and HNC risk. The pooled ORs were determined for an allelic contrast model (C allele vs. G allele), a dominant model (CC+CG vs. GG), and a recessive model (CC vs. CG+GG).

A chi-squared-based Q-statistic test was performed to assess between-study heterogeneity. A *P* value for the Q-test greater than 0.05 indicates absence of heterogeneity, and then the ORs were pooled by a fixed-effect model (Mantel-Haenszel) [28]; otherwise, they were pooled by a random-effect model (DerSimonian and Laird) [29]. The significance of the pooled ORs was determined by Z-test. For evaluation of the publication bias, funnel plots were created. If the plot was asymmetrical, an evident publication bias might exist [30]. To minimize the subjective influence of the visual inspection assessment, we further used Egger's linear regression test to evaluate the symmetry of the funnel plot [31]. All statistical analysis in the present study was performed using the program Microsoft Excel 2003 and STATA 11.0 software (Stata Corporation, Texas, USA).

## ABBREVIATIONS

MiR: MicroRNA
SNP: single nucleotide polymorphism
HNC: head & neck cancer
HB: hospital-based
PB: population-based
OR: Odds Ratio
CI: confidence interval
HWE: Hardy-Weinberg equilibrium
